# Outcomes in elderly patients undergoing endovascular thrombectomy in association with premorbid Rankin Scale scores

**DOI:** 10.3389/fneur.2024.1418415

**Published:** 2024-07-03

**Authors:** Franziska M. Ippen, Katharina Schregel, Matthias Ungerer, Manuel Feisst, Peter A. Ringleb, Christoph K. Gumbinger

**Affiliations:** ^1^Department of Neurology, Heidelberg University Hospital, Heidelberg, Germany; ^2^Department of Neuroradiology, Heidelberg University Hospital, Heidelberg, Germany; ^3^Institute of Medical Biometry, University of Heidelberg, Heidelberg, Germany

**Keywords:** acute ischemic stroke, endovascular thrombectomy, elderly patients, premorbid Rankin Scale score, outcome

## Abstract

**Background:**

Endovascular thrombectomy (EVT) reduces disability in patients with acute ischemic stroke (AIS); however, its efficacy in patients aged >80 years remains unclear.

**Objectives:**

This study aimed to assess the impact of premorbid modified Rankin Scale (pmRS) scores and age on patients with AIS undergoing EVT and the effect of EVT on functional outcome and mortality.

**Methods:**

We conducted a retrospective cohort study and screened the Heidelberg Recanalization Registry (HeiReKa) database for patients with AIS between 1999 and 2021. Outcomes were stratified by age (<80, 80–89, and ≥90 years) and pmRS score (0–2 vs. 3–5). Adjusted odds ratios for outcomes and mortality at 3 months after treatment were examined.

**Results:**

Finally, 2,591 patients were included [including those aged ≥90 years (*n* = 158)]. Poor functional outcomes were associated with advanced age, vascular risk factors, stroke severity, and vessel status. Conversely, lower prestroke disability and younger age were associated with better outcomes and reduced mortality. A pmRS of 3–5 was associated with an increased risk of mortality and worse functional outcomes regardless of age. Notably, patients aged ≥90 years with a pmRS of 0–2 had significantly better outcomes than those aged <80 years with a pmRS of 3–5.

**Conclusion:**

Both age and pmRS are important in assessing the benefits of EVT. However, prestroke functional status might be more crucial than biological age in determining outcomes following EVT.

## Introduction

The incidence of acute ischemic stroke (AIS) increases with age (1), affecting ~30% of patients aged ≥80 years and is expected to increase in the future (1, 2). Studies have demonstrated a positive risk-benefit profile in functional outcomes from intravenous thrombolysis (IVT) in elderly patients; therefore, an upper age limit is no longer formalized in the European and American guidelines (3, 4). Furthermore, a large observational study revealed that IVT is beneficial even in patients with severe preexisting disabilities (premorbid modified Rankin Scale (pmRS) scores) (5).

In randomized clinical trials (RCT) of endovascular thrombectomy (EVT) for AIS, patients aged ≥80 years are often excluded or underrepresented (e.g., due to more clinical trial restrictions). The safety and efficacy of EVT in octogenarians remains controversial (6, 7).

Regarding pmRS, successful recanalization is reportedly the most significant predictor of a beneficial functional outcome (8). Nevertheless, patients with less favorable functional status (a pmRS score of 3–4) appear to benefit less in terms of desirable functional outcomes than those with better previous pmRS scores (0–2) (8). However, a combined assessment of pmRS scores and age has not been conducted when investigating outcomes after EVT in AIS. Therefore, this study aimed to assess the impact of pmRS scores and age on octogenarians and older patients compared to those aged <80 years with large/small vessel occlusion and the effect of EVT on functional outcomes and mortality. We hypothesized that, compared to patients aged <80 years, the functional outcomes after EVT of those aged 80–89 and ≥90 years will likely depend on their pmRS scores at admission.

## Methods

This cross-sectional study included AIS patients aged >18 years with small/large vessel occlusions of the anterior and posterior circulation within and outside the time frame eligible for EVT or IVT immediately followed by EVT. The study was prospectively registered in our consecutive Heidelberg Recanalization Registry (9–11) (HeiReKa) and approved by the Medical Faculty Ethics Committee, the University of Heidelberg (S-325/2015).

Standard descriptive statistics were used to analyze the patients' demographic data. Stroke severity was measured using the National Institutes of Health Stroke Scale (NIHSS) and the modified Rankin Scale (mRS). The mRS score was documented at admission and discharge. A prestroke mRS (pmRS) was estimated at admission. A 3-month functional outcome was assessed by a non-blinded investigator based on outpatient visits, telephone interviews, or discharge reports from rehabilitation units.

We aimed to investigate the association between clinical outcomes 3 months after discharge and patients' age and their pmRS score at admission. Therefore, the study's primary endpoint was defined as a dichotomized mRS score at 3 months. “Favorable outcome” was defined as an mRS score of 0–2 or back to baseline, indicating only limited functional dependency after stroke. “Unfavorable outcome” was defined as an mRS score of 3–6 or worse than at baseline. Considering that elderly patients are more likely to have physical constraints than younger patients, the distribution of every point on the pmRS before and the mRS after the intervention in patients aged <80, 80–89, and >90 years was also assessed and compared.

We used multivariable binary logistic regression analysis to assess the association between functional outcomes, age, and pmRS. In the primary model, we coded the outcome variables as favorable outcome when mRS is 0–2 or back to baseline vs. mRS 3–6; and mortality when mRS is 0–5 vs. mRS 6. After adjustment for potential confounding variables (sex, pmRS, preexisting comorbidities, risk factors, NIHSS at the time of admission, prestroke mRS, medication, and site of vessel occlusion, and the onset to treatment time in the case of a known onset), regression coefficients were used to calculate adjusted odds ratios (ORs) to estimate the association between mRS at discharge and the influence of the patient's age and pmRS at admission. A comparable model was used to calculate ORs for mortality.

All statistical tests were two-sided. The *p*-values of < 0.05 were considered statistically significant in a descriptive sense (exploratory analysis). Data analyses were conducted using R Studio Version 4.0.0 (Posit PBC, Boston, MA) and GraphPad Prism 9 (Dotmatics, Boston, MA).

## Results

### Baseline and procedural characteristics

**Figure 1:**
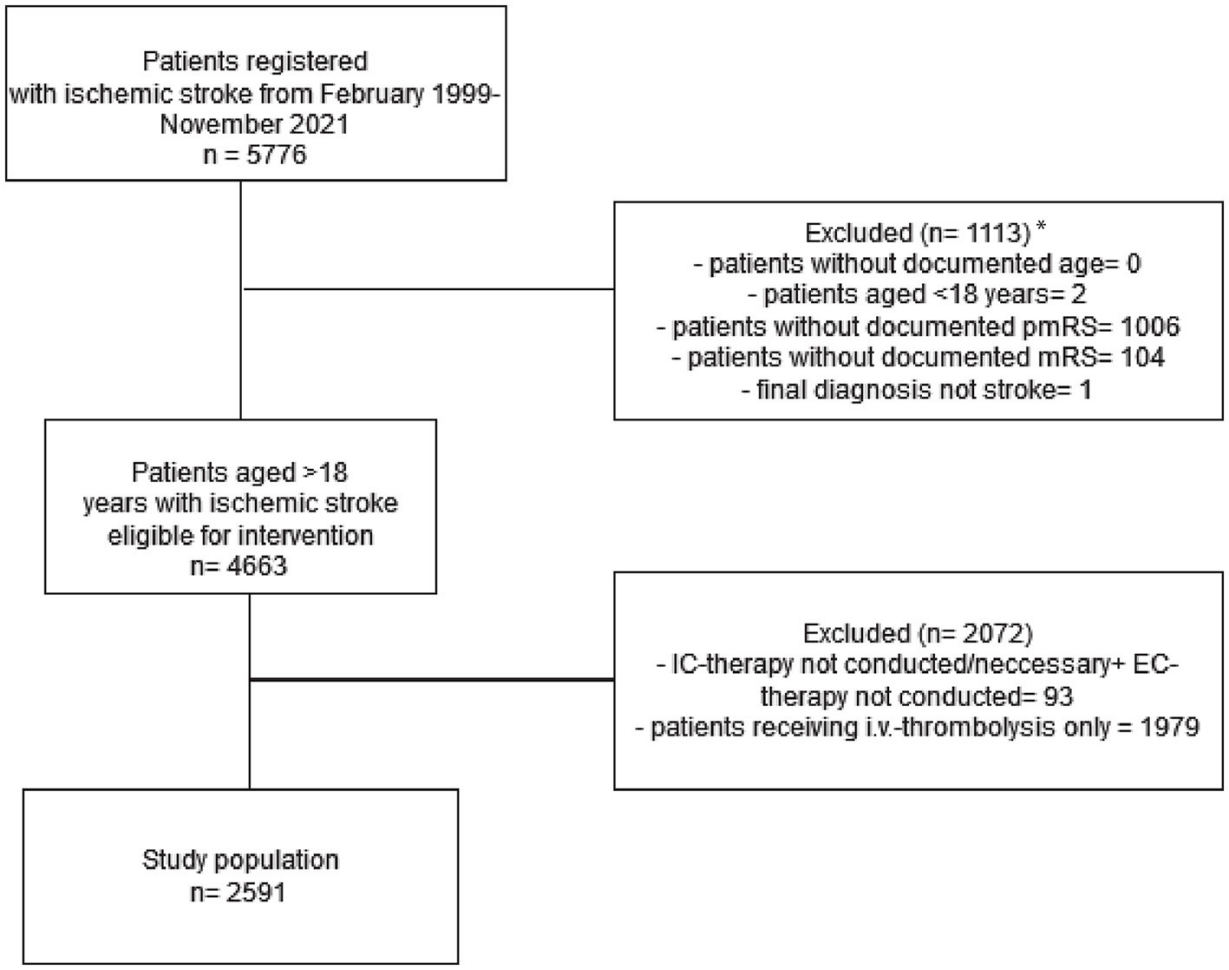
The inclusion and exclusion criteria of the study cohort. *The high amount of undocumented pmRS is mainly explained by patients from external referrers from other healthcare facilities transferred to Heidelberg University Hospital for EVT only. IC, intracranial; EC, extracranial; pmRS, premorbid modified Rankin Scale.

Between 1999 and November 2021, 5,776 patients with suspected AIS received EVT at the University Hospital, Heidelberg. Patients with recanalization before EVT (who received only IVT in most cases), with stroke mimics, who aged < 18 years, and with missing 3-month outcome data were excluded. Of the 5,776 patients, we excluded 1,006 patients due to the lack of documented pmRS scores at admission, and 1,979 patients for having received only i.v. thrombolysis. For a flow chart representation, refer to Figure 1. The main reason for undocumented pmRS was that most patients were referred from external healthcare facilities to Heidelberg University Hospital specifically for EVT.

Finally, 2,591 patients were included in the analysis. These patients were subcategorized according to age and pmRS scores at admission. Men represented 52% of the study population. Furthermore, 81% of patients were admitted with a pmRS score of 0–2, while 19% had a pmRS score of 3–5. Patients aged <80 years (Cohort A, *n* = 1,647) accounted for 64% of the sample, those aged 80–89 years (Cohort B, *n* = 786) accounted for 30%, and patients aged ≥90 years (Cohort C, *n* = 158) comprised 6% of the study cohort. For detailed patient characteristics, refer to Table 1.

**Table 1:** Demographic and clinical characteristics of the patients at baseline.

**Variable**	**Cohort A**		**Cohort B**		**Cohort C**		**Total**	
	<**80 years**		**80–89 years**		≥**90 years**		**(*****n*** = **2,591)**	
	**(*****n*** = **1,647)**		**(*****n*** = **786)**		**(*****n*** = **158)**			
	**pmRS 0–2**	**pmRS 3–5**	**pmRS 0–2**	**pmRS 3–5**	**pmRS 0–2**	**pmRS 3–5**	**pmRS 0–2**	**pmRS 3–5**
	**(*n* = 1,488)**	**(*n* = 159)**	**(*n* = 535)**	**(*n* = 251)**	**(*n* = 71)**	**(*n* = 87)**	**(*n* = 2,094)**	**(*n* = 497)**
Mean age ±SD—yr (min-max)	66 ± 11 (19–79)	71 ± 8.4 (25–79)	83 ± 2.6 (80–89)	84 ± 2.8 (80–89)	92 ± 2.2 (90–99)	92 ± 2.3 (90–100)	71 ± 13 (19–99)	82 ± 9.3 (25–100)
Male sex, *n* (%)	845 (57)	91 (57)	220 (41)	74 (29)	17 (24)	9 (10)	1,082 (52)	174 (35)
Time frame median- min (min-max)	157 (0–1,270)	177 (45–986)	180 (0–1,433)	221 (27–1,249)	152 (36– 1,198)	215 (30– 1,370)	164 (0–1,433)	210 (27–1,370)
Unwitnessed/wake up onset (%)	445 (35)	54 (36)	181 (36)	86 (35)	29 (44)	43 (49)	655 (35)	183 (38)
Median NIHSS at admission (IQR)	15 (9–20)	18 (12–23)	16 (11–21)	17 (11–22)	17 (11– 22)	19 (14– 22)	15 (10–20)	18 (12–22)
Median pmRS at admission (IQR)	0 (0–1)	3 (3–4)	1 (0–2)	3 (3–5)	1 (1–2)	3 (3–3)	0 (0–1)	3 (3–3)
**Previously diagnosed comorbidities and risk factors (** ***n*** **%)**								
Arterial hypertension	1,015 (68)	123 (77)	468 (87)	224 (89)	57 (80)	82 (94)	1,540 (74)	429 (86)
Diabetes	314 (21)	57 (36)	122 (23)	80 (32)	10 (14)	20 (23)	446 (21)	157 (32)
Hypercholesterolemia	488 (33)	74 (47)	198 (37)	103 (41)	21 (30)	29 (33)	707 (34)	206 (42)
PAD	81 (5)	25 (16)	28 (5)	27 (11)	1 (1)	3 (3)	110 (5)	55 (11)
Atrial fibrillation	506 (34)	76 (48)	334 (62)	166 (66)	40 (56)	63 (72)	880 (42)	305 (61)
Coronary heart disease	336 (23)	58 (36)	164 (31)	91 (36)	14 (20)	24 (28)	514 (25)	173 (35)
Current smoking	292 (20)	23 (14)	17 (3)	7 (3)	1 (1)	0 (0)	310 (15)	30 (6)
**Previous medication (** ***n*** **%)**								
Oral anticoagulation	231 (16)	44 (28)	152 (28)	87 (35)	16 (23)	24 (28)	399 (19)	155 (31)
Thrombocyte aggregation inhibition (single/dual APT)	383 (26)/39 (3)	64 (40)/8 (5)	177 (33)/20 (4)	84 (33)/7 (3)	30 (42)/3 (4)	29 (33)/1 (1)	590 (28)/62 (3)	177 (36)/16 (3)
Statin	405 (28)	63 (41)	173 (33)	87 (36)	19 (28)	23 (27)	597 (30)	173 (36)
**Brain imaging characteristics (** ***n*** **%)**								
ASPECTS median (min-max)	9 (0–10)	9 (1–10)	9 (0–10)	9 (3–10)	9 (2–10)	9 (4–10)	9 (0–10)	9 (1–10)
**Localization of ischemic stroke (** ***n*** **%)**								
Supratentorial	1,312 (88)	143 (90)	479 (90)	227 (90)	65 (92)	84 (97)	1,856 (89)	454 (91)
Infratentorial	176 (12)	16 (10)	56 (10)	24 (10)	6 (8)	3 (3)	238 (11)	43 (9)
**Site of vessel occlusion (** ***n*** **%)**								
M1/ACA/PCA	573 (39)	63 (40)	231 (43)	109 (43)	32 (45)	47 (54)	836 (40)	219 (44)
M2/M1M2/M3	247 (17)	32 (20)	87 (16)	57 (23)	14 (20)	12 (14)	348 (17)	101 (20)
ICA/ICA-MCA/ICA-T	494 (33)	48 (30)	163 (30)	61 (24)	19 (27)	25 (29)	676 (32)	134 (27)
BA/VA	174 (12)	16 (10)	54 (10)	24 (10)	6 (8)	3 (3)	234 (11)	43 (9)

SD, standard deviation; pmRS, premorbid modified Rankin Scale; NIHSS, National Institutes of Health Stroke Scale; ASPECTS, Alberta Stroke Program Early CT Score; ICA, internal carotid artery; MCA, middle cerebral artery; M1, middle cerebral artery, segment 1; M2, middle cerebral artery, segment 2; M3, middle cerebral artery, segment 3; ACA, anterior cerebral artery; BA, basilar artery; VA, vertebral artery; PCA, posterior cerebral artery; APT, antiplatelet therapy; IQR, interquartile range; and PAD, peripheral arterial occlusive disease.

Both recanalization results and bleeding complications showed no correlations with respect to age or pmRS scores. However, patients with a pmRS score of 3–5 showed a higher d90 mortality rate. For details, refer to Table 2.

**Table 2:** Procedural characteristics and outcome parameters of the study cohort.

**Variable**	**Cohort A**		**Cohort B**		**Cohort C**		**Total**	
	<**80 years**		**80–89 years**		≥**90 years**		**(*****n*** = **2,591)**	
	**(*****n*** = **1,647)**		**(*****n*** = **786)**		**(*****n*** = **158)**			
**Treatment characteristics**								
i.v. thrombolysis (%)	838 (56)	62 (39)	254 (47)	87 (35)	30 (42)	43 (49)	1,122 (54)	192 (39)
i.a. thrombolysis (%)	650 (44)	97 (61)	281 (53)	164 (65)	41 (58)	44 (51)	972 (46)	305 (61)
Median DNT (min; IQR)	36 (26–55)	40 (29–55)	36 (28–55)	36 (26–59)	40 (30–66)	36 (31–45)	36 (27–55)	36 (28–54)
Median DVT (min; IQR)	75 (56–97)	73 (56–96)	75 (56–97)	72 (56–95)	74 (64–102)	74 (57–99)	75 (56–97)	73 (57–96)
**EC therapy**								
- Nothing	1,180 (80)	134 (85)	473 (89)	230 (92)	65 (93)	84 (98)	1,718 (83)	448 (91)
- Stent	270 (18)	24 (15)	51 (10)	18 (7)	5 (7)	2 (2)	326 (16)	44 (9)
- PTA	31 (2)	0 (0)	7 (1)	1 (0)	0 (0)	0 (0)	38 (2)	1 (0)
**Recanalization (TICI/analog TICI), (** ***n*** **%)**								
- TICI 3	611 (41)	62 (39)	222 (42)	106 (42)	29 (41)	41 (47)	862 (41)	209 (42)
- TICI 2c	182 (12)	18 (11)	65 (12)	28 (11)	8 (11)	14 (16)	255 (12)	60 (12)
- TICI 2b	405 (27)	46 (29)	147 (28)	55 (22)	18 (25)	14 (16)	570 (27)	115 (23)
- TICI 2a	93 (6)	8 (5)	36 (7)	10 (4)	4 (6)	4 (5)	133 (6)	22 (4)
- TICI 1	19 (1)	2 (1)	5 (1)	2 (1)	2 (3)	2 (2)	26 (1)	6 (1)
- TICI 0	129 (9)	20 (13)	48 (9)	46 (18)	9 (13)	12 (14)	186 (9)	78 (16)
- Unclear/missing information	49 (3)	3 (2)	11 (2)	3 (1)	1 (1)	0 (0)	61 (3)	6 (1)
**Bleeding complications (HBC)**								
- No ICH	1,052 (71)	110 (70)	383 (72)	185 (76)	53 (75)	69 (82)	1,488 (71)	364 (75)
- Class 1	275 (19)	25 (16)	97 (18)	43 (18)	13 (18)	11 (13)	385 (18)	79 (16)
- Class 2	80 (5)	6 (4)	25 (5)	5 (2)	2 (3)	0 (0)	107 (5)	11 (2)
- Class 3	76 (5)	15 (10)	24 (5)	12 (5)	3 (4)	4 (5)	103 (5)	31 (6)
- Other/missing information	0 (0)	1 (1)	1 (0)	0 (0)	0 (0)	3	1 (0)	0 (0)
**Functional outcome**								
- mRS 0	139 (9)	0 (0.0)	20 (4)	0 (0)	3 (4)	1 (1)	162 (8)	1 (0)
- mRS 1	242 (16)	0 (0.0)	44 (8)	1 (0)	3 (4)	1 (1)	162 (8)	2 (0)
- mRS 2	298 (20)	5 (3)	76 (14)	6 (2)	6 (8)	1 (1)	380 (18)	12 (2)
- mRS 3	242 (16)	34 (21)	109 (20)	51 (20)	6 (8)	11 (13)	364 (17)	96 (19)
- mRS 4	213 (14)	25 (16)	81 (15)	56 (22)	11 (15)	17 (20)	305 (15)	98 (20)
- mRS 5	101 (7)	20 (13)	34 (6)	20 (8)	9 (13)	5 (6)	144 (7)	45 (9)
- mRS 6	253 (17)	75 (47)	171 (32)	117 (47)	26 (37)	51 (59)	450 (21)	243 (49)

i.v., intravenous; DNT, door-to-needle time; DVT, door-to-vessel time; i.a., intraarterial; ICH, intracranial hemorrhage; TICI, thrombolysis in cerebral infarction score; HBC, Heidelberg Bleeding Classification; mRS, modified Rankin Scale; IQR, interquartile range.

### Favorable outcome

**Figure 2:**
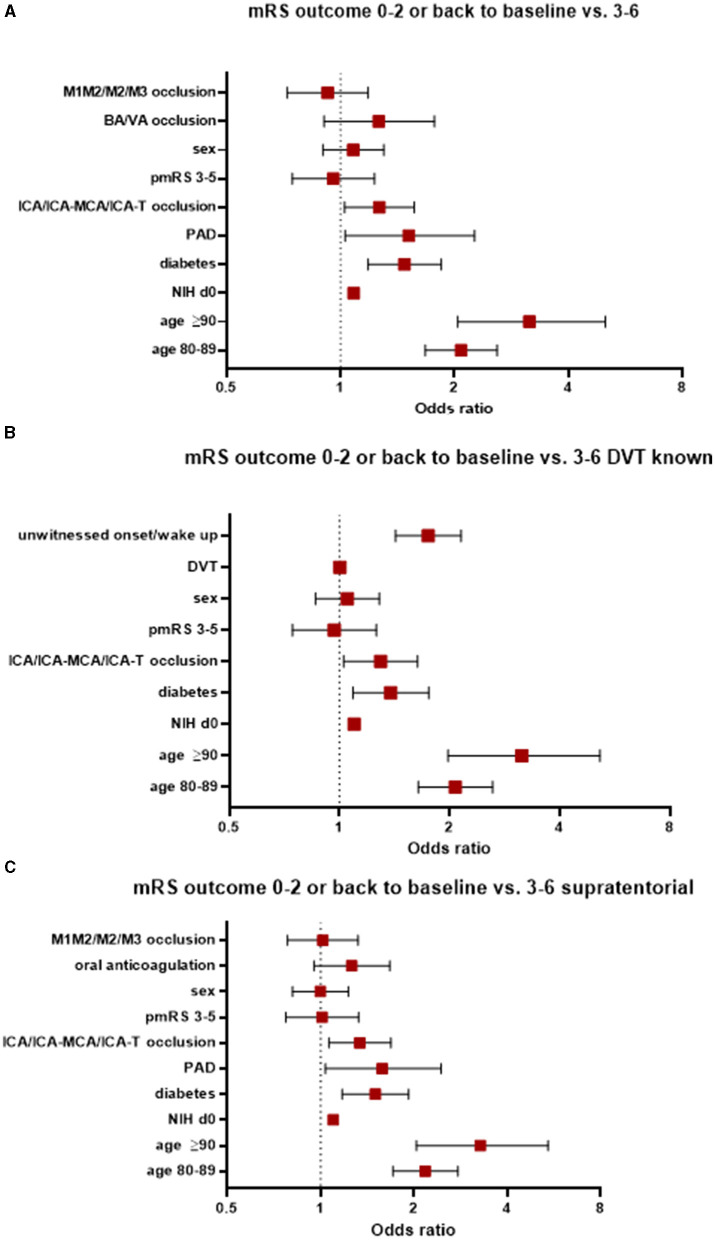
Factors associated with worse functional outcomes **(A)** in the entire study population, **(B)** those with known DVT status, and **(C)** those with supratentorial AIS. AIS, acute ischemic stroke; pmRS, premorbid modified Rankin Scale; ICA, internal carotid artery; MCA, middle cerebral artery; M1, middle cerebral artery, segment 1; M2, middle cerebral artery, segment 2; M3, middle cerebral artery, segment 3; BA, basilar artery; VA, vertebral artery; DVT, door-to-vessel time; NIH d0, National Institutes of Health Stroke Scale at admission; PAD, peripheral arterial occlusive disease.

Patients with an mRS score of 0–2 at 90 days or whose mRS scores were the same as at baseline compared favorably to those whose d90-mRS scores deteriorated to 3–6. Factors significantly associated with a worse functional outcome included advanced age, the presence of peripheral arterial occlusive disease (PAD, OR 1.52, 95% CI 1.03–2.26), diabetes (OR 1.48, 95% CI 1.18–1.85), occlusion of the internal carotid artery (ICA) alone or at the ICA bifurcation (ICA-T) or in combination with the middle cerebral artery (MCA; ICA/ICA-MCA/ICA-T; OR 1.27, 95% 1.03–1.57), and a worse NIHSS score at admission (OR 1.08, 95% CI 1.07–1.1); see Figure 2A. Notably, better recanalization status was significantly associated with improved outcomes, as evidenced by thrombolysis in cerebral infarction (TICI) scores of 2b (OR 0.26, 95% 0.16–0.40), 2c (OR 0.15, 95% 0.09–0.24), and 3 (OR 0.11, 95% CI 0.07–0.17).

When focusing on patients with a known door-to-vessel time (DVT) and patients with favorable mRS scores compared to patients with an unfavorable mRS score, the following parameters were associated with a worse functional outcome: aged ≥90 years (OR 3.15, 95% CI 1.98–5.15), aged 80–89 years (OR 2.07, 95% CI 1.64–2.62), an unwitnessed onset/wake up stroke (OR 1.75, 95% CI 1.42-2.15), the presence of diabetes (OR 1.38, 95% CI 1.09–1.75), occlusion of the ICA/ICA-MCA/ICA-T (OR 1.3, 95% CI 1.03–1.63), and a worse NIHSS score at admission (OR 1.1, 95% CI 1.08–1.11). See Figure 2B.

In patients with supratentorial AIS, factors associated with a worse functional outcome (0–2 or back to baseline compared to a mRS score of 3–6 at 90 days) were patients aged ≥90 years, aged 80–89 years, with the presence of diabetes, with PAD, with occlusion of the ICA/ICA-MCA/ICA-T, and with a worse NIHSS score at admission (Figure 2C).

### Mortality

**Figure 3:**
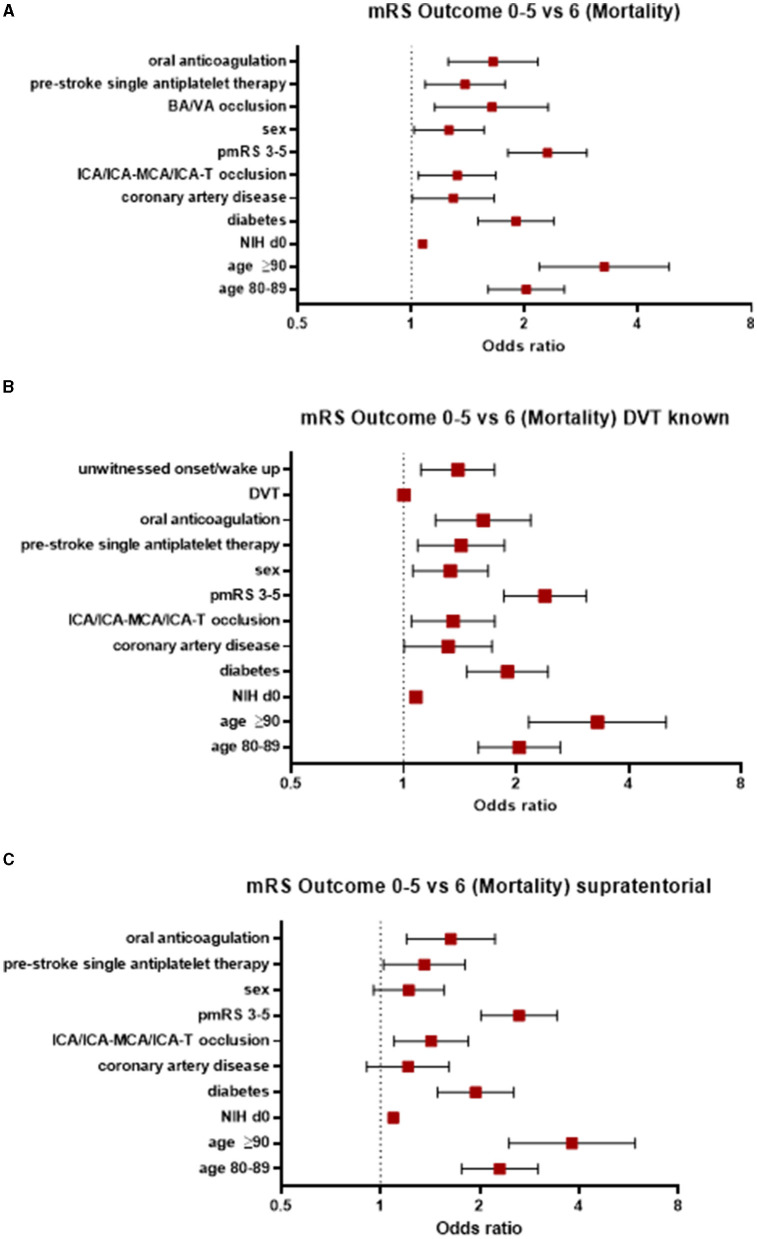
Factors associated with an increased risk of mortality **(A)** in the entire study population, **(B)** those with known DVT status, and **(C)** those with supratentorial AIS. AIS, acute ischemic stroke; pmRS, premorbid modified Rankin Scale; ICA, internal carotid artery; MCA, middle cerebral artery; BA, basilar artery; VA, vertebral artery; DVT, door-to-vessel time; NIH d0, National Institutes of Health Stroke Scale at Admission.

An overall mortality at 90 days was 27% across the entire cohort. In contrast to morbidity, more factors were associated with an increased risk of mortality in AIS patients treated with EVT (see Figure 3A). A better recanalization result was significantly associated with an improved outcome (TICI 2b OR 0.34, 95% 0.23–0.5; TICI 2c OR 0.22, 95% 0.14–0.34; and TICI 3 OR 0.17, 95% 0.11–0.24).

When focusing on patients with a known DVT, the factors mentioned above were significantly associated with an increased risk of mortality, in addition to CAD (OR 1.32, 95% CI 1.0–1.72) and an unwitnessed onset/wake-up stroke (OR 1.4, 95% CI 1.11–1.75). However, DVT itself was not associated with mortality (OR 1.0, 95% CI 1.0–1.0; Figure 3B).

In patients with supratentorial AIS, the parameters associated with an increased risk of mortality were age ≥90 years (OR 3.81, 95% CI 2.45–5.91), a pmRS score of 3–5 (OR 2.63, 95% CI 2.02–3.43), age between 80 and 89 years (OR 2.3, 95% CI 1.76–3.0), the presence of diabetes (OR 1.94, 95% CI 1.49–2.53), oral anticoagulation (OR 1.63, 95% CI 1.2–2.22), prestroke single APT (OR 1.36, 95% 1.02–1.8), ICA/ICA-MCA/ICA-T occlusion (OR 1.42, 95% CI 1.1–1.85), and a NIHSS score at admission (OR 1.09, 95% CI 1.07–1.11; Figure 3C).

### Comparison of subgroups

**Figure 4:**
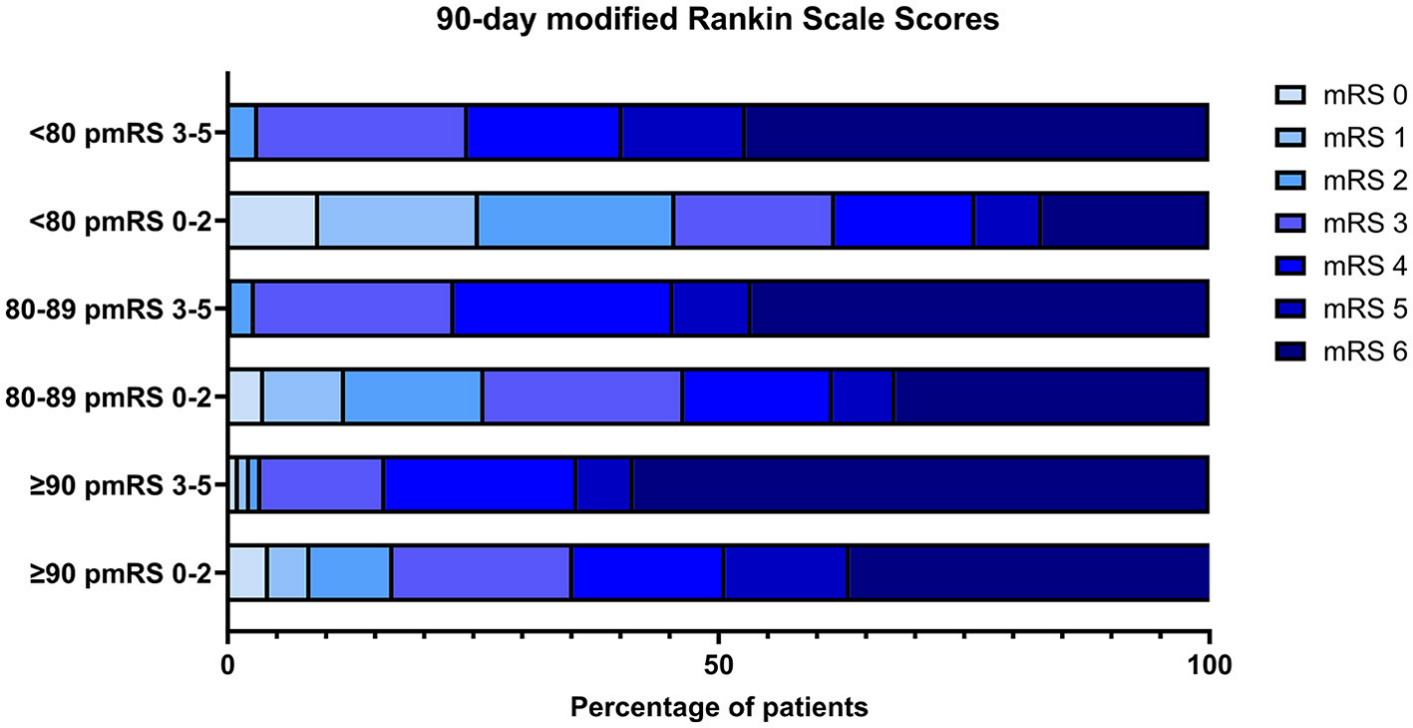
The distribution of mRS at 90 days is stratified by initial pmRS and age group. pre mRS, premorbid modified Rankin Scale; mRS, modified Rankin Scale.

The distribution of scores on the mRS at 90 days corroborated the findings of increased morbidity and mortality with a higher pmRS score at admission (Figure 4).

#### Morbidity

Age and pmRS scores were analyzed with respect to the following outcome cohort: an mRS score of 0–2 or back to baseline vs. an mRS score of 3–6 (Supplementary material 1).

We first compared different age groups within the same initial pmRS subgroup to assess potential outcome differences. When focusing on a pmRS score of 0–2, we observed differences between patients aged <80 and 80–89/≥90 years, indicating an improved outcome for younger patients. However, we did not see these differences between patients aged 80–89 and ≥90 years. A comparison of all age groups showed no differences in the pmRS score of 3–5.

Furthermore, a comparison of different pmRS subgroups within the same age group showed a difference in outcome for patients aged <80 years with a pmRS score of 0–2 vs. that of 3–5, favoring the cohort with better initial pmRS scores.

Regarding a favorable outcome in patients aged <80 years with a pmRS score of 0–2 vs. those aged 80–89/≥90 years with a pmRS score of 3–5, the results favored the younger subgroup with better pmRS scores at admission. Significant differences regarding an improved outcome were observed when comparing patients aged ≥90 years with a pmRS score of 0–2 to those aged <80 years with a pmRS score of 3–5.

#### Mortality

Significant differences in mortality were obtained when patients aged <80 vs. 80–89/≥90 years with a pmRS score of 0–2 and those aged 80–89 vs. ≥90 years with a pmRS score of 3–5, indicating that a younger age at the same functional level might account for beneficial outcomes after the intervention.

All age subgroups showed significant differences in mortality considering their individual pmRS scores at baseline. No differences in mortality were found when analyzing patients aged ≥90 years with pmRS scores of 0–2 vs. those aged <80/80–89 years with pmRS scores of 3–5.

A comparison between patients aged <80/80–89 years with pmRS scores of 0–2 and all other age groups with pmRS scores of 3–5 showed significant differences in mortality, indicating that a better functional status at admission in patients aged <80 and 80–89 years and is associated with a decreased risk of mortality. The results are displayed in Supplementary material 2.

## Discussion

Our findings showed that advanced age of >80 years, particularly ≥90 years, vascular risk factors, the magnitude of assessed NIHSS scores at admission, major vessel occlusion of the anterior circulation, and either known DVT or an unwitnessed onset/wake-up stroke were associated with an increased risk for unfavorable outcomes and mortality after EVT. Although a preexisting unfavorable functional status (pmRS scores of 3–5) did not increase the risk of unfavorable outcomes, it was associated with an increased risk of mortality. The use of prestroke medications, such as single antiplatelet therapy (APT) and oral anticoagulation, CAD, and occlusion of the posterior circulation (BA/VA), was associated with an increased risk of mortality.

Conversely, we found that if a good functional status (pmRS 0–2) was present at baseline, younger age was associated with an improved outcome and a decreased risk of mortality. However, for patients with a pmRS score of 3–5, this status was associated with an increased risk of mortality and showed no advantage related to younger age for an improved functional outcome. Moreover, in patients aged >80 years, differences in pmRS scores showed no difference in functional outcomes. Notably, patients aged ≥90 years with a pmRS score of 0–2 had a significantly better outcome than those aged <80 years with a pmRS score of 3–5. Better recanalization results were significantly associated with improved outcomes and lower risk of mortality. These findings are in line with previous studies (12–15).

To the best of our knowledge, this is the first study to compare the outcomes of EVT in patients across various age groups in combination with their pmRS scores. Previous studies primarily focused on either pmRS scores or age independently as factors influencing outcomes after EVT (8, 12, 16–18). However, this is challenging in daily clinical emergency settings because a good functional status might favor the decision of intervention even though the patient is at an advanced age or vice versa. Our results indicated that younger patients with AIS are likely to benefit the most from EVT. Notably, patients aged ≥90 years with better pmRS scores had better functional outcomes after intervention than those aged <80 years with worse pmRS scores at admission. These findings indicate that the most crucial factor in terms of clinical outcome might be the extent of preexisting disabilities rather than the biological age of the patient. However, age remains an important factor. Within the three rather homogeneous age subgroups, a poor pmRS score at baseline also appeared to be the major predictor of mortality.

In 2015, five RCTs demonstrated that AIS patients with the occlusion of the proximal anterior circulation treated with EVT demonstrated significantly reduced disability at 90 days compared to those receiving standard medical care (SMC) (19–23). A subsequent meta-analysis of these trials showed that improvements in these patient characteristics, including individual characteristics, such as advanced age (including octogenarians) (12), thereby favor the use of EVT even in elderly patients.

More recently, several RCTs have shown that AIS patients with occlusion in the anterior circulation and a large ischemic core, treated within 24 h of onset, achieved better functional outcomes at 90 days after admission compared to SMC, maintaining an acceptable safety profile (24–27). Notably, one of these trials showed that these results also applied to elderly patients aged ≥ 70 years old (25). The findings of these studies demonstrate the need for a clinically based rather than an imaging-based selection of patients for EVT, further highlighting the relevance of our investigation.

The strengths of our study include the comprehensive data and large sample size from the HeiReKa recanalization registry, enabling the generalizability of our results and making an adjustment for potential confounding variables possible.

However, the limitations of this study include the selection bias of patients included in the EVT cohort, which is inherent due to its retrospective nature of this study. Patients with major functional constraints from preexisting disabilities may have either not been transferred to Heidelberg University Hospital (for example, due to an advance directive, etc.) or may have been primarily admitted to community hospitals instead (28). Consequently, the rate of withdrawal of care is undocumented in the registry. Mortality analyses could be prone to selection bias because patients with worse pmRS scores (e.g., those bedridden at stroke onset) are more likely to receive the best supportive care, which might have an impact on the outcome analysis. Furthermore, other relevant data that could impact our understanding of either EVT itself, such as rates of mechanical ventilation, or the pmRS, such as the cause of premorbid disability, and referrals to rehabilitation after EVT, are not covered in the HeiReKa registry and were therefore not assessed in the analysis.

## Conclusion

Our findings suggest a combined approach to decision-making in treating AIS with EVT, emphasizing the importance of considering both age and pmRS scores in order to derive a potential benefit of an EVT for affected patients with AIS. Our results indicate that pmRS is more important than age.

## Data availability statement

The raw data supporting the conclusions of this article will be made available by the authors, without undue reservation.

## Ethics statement

The studies involving humans were approved by Medical Faculty Ethics Committee, University of Heidelberg. The studies were conducted in accordance with the local legislation and institutional requirements. Written informed consent for participation was not required from the participants or the participants' legal guardians/next of kin because the study was prospectively registered in our consecutive Heidelberg Recanalization Registry (9–11) (HeiReKa) and approved by the Medical Faculty Ethics Committee, University of Heidelberg (S-325/2015).

## Author contributions

FI: Writing – original draft, Visualization, Methodology, Investigation, Formal analysis, Data curation, Conceptualization. KS: Writing – review & editing, Visualization, Software, Methodology, Formal analysis. MU: Writing – review & editing, Visualization, Validation. MF: Writing – review & editing, Validation, Software, Resources, Methodology, Formal analysis. PR: Writing – review & editing, Validation, Resources, Project administration, Data curation. CG: Writing – review & editing, Visualization, Validation, Supervision, Resources, Project administration, Methodology, Investigation.
