# Supplementary material for: Outcomes in elderly patients undergoing endovascular thrombectomy in association with premorbid Rankin Scale scores
Source: Front Neurol. 2024 Jul 3;15:1418415. doi: 10.3389/fneur.2024.1418415 (PMC11252042; doi:10.3389/fneur.2024.1418415)
Supplement: Supplementary file 1 [file Data_Sheet_1.docx]

**Supplementary File 1**

Outcome Comparisons: Morbidity

**Comparison of different age groups within the same initial pmRS**

**Outcome mRS 0–2 or back to baseline versus 3–6**

**pmRS 0–2**

pmRS 0–2 (<80 vs. 80–90 years): outcome 0–2 or back to baseline versus 3–6

p<0.0001

pmRS 0–2 (<80 vs. >90 years): outcome 0–2 or back to baseline versus 3–6

p<0.0001

pmRS 0–2 (80–90 vs. >90 years): outcome 0–2 or back to baseline versus 3–6

p=0.1086

**pmRS 3–5**

pmRS 3–5 (<80 vs. 80–90 years): outcome 0–2 or back to baseline versus 3–6

p=0.6519

pmRS 3–5 (<80 vs. >90 years): outcome 0–2 or back to baseline versus 3–6

p=0.1732

pmRS 3–5 (80–90 vs. >90 years): outcome 0–2 or back to baseline versus 3–6

p=0.2565

**Comparison of different pmRS groups within the same initial age**

**Outcome mRS 0–2 or back to baseline versus 3–6**

<80 years (pmRS 0–2 vs. 3–5): outcome 0–2 or back to baseline versus 3-6

p=0.0003

80–90 years (pmRS 0–2 vs. 3–5): outcome 0–2 or back to baseline versus 3–6

p=0.7951

>90 years (pmRS 0–2 vs. 3–5): outcome 0–2 or back to baseline versus 3–6

p=0.6840

**Comparison of different age and pmRS groups**

**Outcome mRS 0–2 or back to baseline versus 3–6**

<80 years, pmRS 0–2 versus 80–90 years, pmRS 3–5: outcome 0–2 or back to baseline versus 3–6

p<0.0001

<80 years, pmRS 0–2 versus >90 years, pmRS 3–5: outcome 0–2 or back to baseline versus 3–6

p<0.0001

80–90 years, pmRS 0–2 versus <80 years, pmRS 3–5: outcome 0–2 or back to baseline versus 3–6

p=0.4160

80–90 years, pmRS 0–2 versus >90 years, pmRS 3–5: outcome 0–2 or back to baseline versus 3–6

p=0.3523

>90 years, pmRS 0–2 versus <80 years, pmRS 3–5: outcome 0–2 or back to baseline versus 3–6

p=0.0499

>90 years, pmRS 0–2 versus 80–90 years, pmRS 3–5: outcome 0–2 or back to baseline versus 3–6

p=0.0882
