# Supplementary material for: Outcomes in elderly patients undergoing endovascular thrombectomy in association with premorbid Rankin Scale scores
Source: Front Neurol. 2024 Jul 3;15:1418415. doi: 10.3389/fneur.2024.1418415 (PMC11252042; doi:10.3389/fneur.2024.1418415)
Supplement: Supplementary file 2 [file Data_Sheet_2.docx]

**Supplementary File 2**

Outcome Comparisons: Mortality

**Comparison of different age groups within the same initial pmRS**

**Outcome mRS 0–5 vs. 6**

**pmRS 0–2**

pmRS 0–2 (<80 vs. 80–90 years): outcome 0–5 vs. 6

p<0.0001

pmRS 0–2 (<80 vs. >90 years): outcome 0–5 vs. 6

p=0.0003

pmRS 0–2 (80–90 vs. >90 years): outcome 0–5 vs. 6

p=0.4223

**pmRS 3–5**

pmRS 3–5 (<80 vs. 80–90 years): outcome 0–5 vs. 6

p=0.9195

pmRS 3–5 (<80 vs. >90 years): outcome 0–5 vs. 6

p=0.1092

pmRS 3–5 (80–90 vs. >90 years): outcome 0–5 vs. 6

p=0.0621

**Comparison of different pmRS groups within the same initial age**

**Outcome mRS 0–5 vs. 6**

<80 years (pmRS 0–2 vs. 3–5): outcome 0–5 vs. 6

p<0,0001

80–90 years (pmRS 0–2 vs. 3–5): outcome 0–5 vs. 6

p<0.0001

>90 years (pmRS 0–2 vs. 3–5): outcome 0–5 vs. 6

p=0.0068

**Comparison of different age and pmRS groups**

**Outcome mRS 0–5 vs. 6**

<80 years, pmRS 0–2 vs. 80–90 years, pmRS 3–5: outcome 0–5 vs. 6

p<0.0001

<80 years, pmRS 0–2 vs. >90 years, pmRS 3–5: outcome 0–5 vs. 6

p<0.0001

80–90 years, pmRS 0–2 vs. <80 years, pmRS 3–5: outcome 0–5 vs. 6

p=0.0006

80–90 years, pmRS 0–2 vs. >90 years, pmRS 3–5: outcome 0–5 vs. 6

p<0.0001

>90 years, pmRS 0–2 vs. <80 years, pmRS 3–5: outcome 0–5 vs. 6

p=0.1520

>90 years, pmRS 0–2 vs. 80–90 years, pmRS 3-5: outcome 0–5 vs. 6

p=0.1397
